# Analysis of pathogenic characteristics of acute respiratory tract infections in children and the influence of environmental factors in Shijiazhuang area

**DOI:** 10.3389/fcimb.2026.1725358

**Published:** 2026-04-24

**Authors:** Shangze Liu, Guangyue Han, Caixiao Jiang, Minghao Geng, Wentao Wu, Nana Guo, Xu Han, Yan Li, Qi Li

**Affiliations:** 1 School of Public Health, Hebei Medical University, Shijiazhuang, Hebei, China; 2 Hebei Provincial Center for Disease Control and Prevention, Shijiazhuang, Hebei, China

**Keywords:** acute respiratory tract infection, DLNM, meteorological factors, multiple pathogens, popular characteristics

## Abstract

**Objective:**

To investigate the epidemiological patterns of acute respiratory infections (ARI) in children (0–14 years) in Shijiazhuang and the impact of meteorological factors.

**Methods:**

Fourteen respiratory pathogens were detected by fluorescent PCR melting curve analysis. LASSO regression screened environmental factors; generalized additive model (GAM) analyzed nonlinear associations between meteorological conditions and ARI incidence; distributed lag nonlinear model (DLNM) explored lagged/cumulative effects of meteorological factors and pollutants. Pathogen distribution differences were tested by χ² or Fisher’s exact test.

**Results:**

A total of 1,961 ARI cases were included, with an overall pathogen positivity rate of 78.23%. Positivity rates differed significantly by age, season, and case classification (all P < 0.05), being highest in 3–6-year-olds, summer, and outpatient/emergency cases. Single infections (59.19%) were dominant; SP and Hi were prone to mixed infections, EV to quadruple+ infections. Pathogen spectrum varied by age and season. Time-series analysis showed seasonal ARI peaks in winter. DLNM revealed nonlinear associations between all environmental factors and ARI: O_3_ (33.5 μg/m³, RR = 4.3051) and SO_2_ (39.4 μg/m³) had strong effects; high CO (49.40 mg/m³), high temperature (31.1 °C), and low-moderate humidity (27%–42.5%) increased risk; high NO_2_ had moderate lagged risk. GAM confirmed nonlinear associations, with highest ARI risk at 3.63 °C (RR = 1.89, 95% CI:1.74–2.05).

**Conclusion:**

High pathogen detection rate was observed in 2024–2025. ARI had distinct age/season patterns, with winter peaks. Low/high temperature, low humidity, and high SO_2_ were key risk factors with immediate + cumulative effects, providing data for prevention and early warning.

## 1 Introduction

Acute respiratory infection (ARI) is one of the most common diseases in children, including upper respiratory tract infection and lower respiratory tract infection. Although most of these infections are self-limiting, they have an acute onset and strong contagiousness, which can have a certain impact on quality of life. The concentrated occurrence of such infections can also bring certain pressure to hospital diagnosis and management (GBD 2021 Lower Respiratory Infections and Antimicrobial Resistance Collaborators, 2024). Shijiazhuang is the capital of Hebei Province in China, located in the hinterland of the North China Plain and the foot of the Taihang Mountains. It is an important transportation hub in the Beijing-Tianjin-Hebei region. With a total area of approximately 15,800 square kilometers, it has a warm temperate continental monsoon climate. As of the end of 2024, the city’s permanent population was approximately 11,246,600, with an urbanization rate of 72.66%. The Han ethnicity is the main population, and the population is mainly concentrated in the central urban area and the eastern plains. Studies have shown that environmental factors have a certain impact on the incidence and transmission of respiratory diseases (Abrescia et al., 2025). ARI is mostly caused by viral and bacterial infections. Common viruses include influenza virus (influenza virus, Flu), severe acute respiratory syndrome coronavirus 2 (severe acute respiratory syndrome coron-avirus 2, SARS-CoV-2), respiratory syncytial virus (respiratory syncytial virus, RSV), etc., and common bacterial pathogens include Streptococcus pneumonia (streptococcus pneumonia, SP), Haemophilus influenzae (Haemophilus influenzae, Hi), Mycoplasma pneumonia (mycoplasma pneumonia, MP), etc. The clinical manifestations are similar to those of influenza, causing various types of bronchitis, bronchitis and pneumonia (Tabatabai et al., 2023). The population is generally susceptible, and it often causes severe cases in infants and children (GBD 2019 LRI Collaborators, 2022). The distributed lag non-linear model (distributed lag non-linear models, DLNM) simultaneously fits the nonlinear relationship of exposure-response and the lag effect of exposure factors, which can more comprehensively and accurately reflect the health effects of environmental factors (Almon, 1965; Armstrong, 2006; Gasparrini et al., 2010). By quantifying the impact of environmental factors on ARI, establishing and improving the monitoring and early warning mechanism for children’s respiratory diseases can not only provide target values for pollutant control, but also provide scientific basis for formulating preventive measures.

## 2 Objects and methods

### 2.1 Research object

This study collected data on the incidence of ARI in the Shijiazhuang area from 2024 to 2025 and collected relevant cases for testing, as follows: In accordance with the provisions of the International Classification of Diseases, 10th Revision (ICD-10) (World Health Organization, 2023) and the “Technical Guidelines for Sentinel Surveillance of Acute Respiratory Infections,” 1,961 pediatric patients (aged 0–14 years) with acute respiratory infections (ARI) meeting the case criteria were screened at Hebei Provincial Children’s Hospital between January 1, 2024, and December 31, 2025 (ARI cases included influenza-like illness [ILI] cases from outpatient and emergency departments and severe acute respiratory infection [SARI] cases requiring hospitalization); Data on the incidence of acute respiratory infections among children aged 0–14 years in Shijiazhuang City from January 1, 2024, to December 31, 2025, were collected from the Infectious Disease Information Reporting Management System of the Hebei Provincial Center for Disease Control and Prevention; Meteorological data for the same period (January 1, 2024, to December 31, 2025) were obtained from the China Meteorological Administration website (https://www.weather.com.cn/), including average temperature (temp), relative humidity (rhum), atmospheric pressure (AP), and wind speed (WS); Additionally, air pollution data for the period from January 1, 2024, to December 31, 2025, were collected from the historical data of the China Air Quality Monitoring and Analysis Platform (https://www.aqistudy.cn/historydata/), with indicators including PM2.5, PM10, SO2, NO2, CO, and O3. Furthermore, this study has been approved by the Ethics Committee of the Hebei Provincial Center for Disease Control and Prevention, with approval number HeBIRBS2023-001.

### 2.2 Testing method

Collect throat swab or bronchoalveolar lavage fluid samples from ARI patients within 3 days of symptom onset. All samples must be properly stored at 2–8 °C after collection and transported via cold chain to a network laboratory. The network laboratory uses the Berger Multiplex Respiratory Pathogen Nucleic Acid Detection Kit and employs the fluorescent PCR melting curve method to test for Flu, SARS-CoV-2, RSV, HI, MP, SP, rhinovirus (RV), human parainfluenza virus (HPIV), human metapneumovirus (HMPV), coronavirus (HCoV), adenovirus (ADV), bocavirus (HBoV), enterovirus (EV), and Group A Streptococcus (GAS). A result is considered positive when the cycle threshold (CT) is <38.

### 2.3 Statistical analysis

Data were organized using Excel 2021. The Shapiro-Wilk test was used to assess the normality of the quantitative data. Neither the air pollutant indicators nor the meteorological indicators followed a normal distribution; therefore, they were described using the M(P25, P75) distribution. Variable selection was performed using LASSO regression via the “glmnet” package in R. A generalized linear model was used to analyze the relationship between the selected meteorological factors and the daily number of cases. The daily number of cases was set as the dependent variable, with meteorological factors and pollutants serving as exposure factors, while controlling for the “day-of-the-week effect.” Since the distribution of the daily number of cases approximated a Poisson distribution, a regression model was established using the log-link function corresponding to the Poisson distribution. R version 4.5.2 and the “dlnm” package were used to construct the DLNMS (Peng et al., 2020; Zhang et al., 2023). Due to the small sample size, the DLNM was adjusted to ensure basic model performance while avoiding overfitting by reducing the degrees of freedom of the cross-basis functions, optimizing model convergence parameters, simplifying the temperature prediction sequence, and focusing on key quantile analysis. Parameter selection was based on the principle of minimizing the quasi-Akaike information criterion (QAIC) and previous research (Zhao et al., 2020); the reference value for calculating the relative risk (RR) was set as the median of each variable. All predictive models used the median as a reference to calculate the effects for the current day, lags of 1–21 days, and cumulative effects. Chi-square tests or Fisher’s exact test were used to statistically analyze the distribution and epidemiological trends of different pathogen infections across different age groups and seasons. The significance level was set at α = 0.05.

## 3 Results

### 3.1 General characteristics

**Figure 1:**
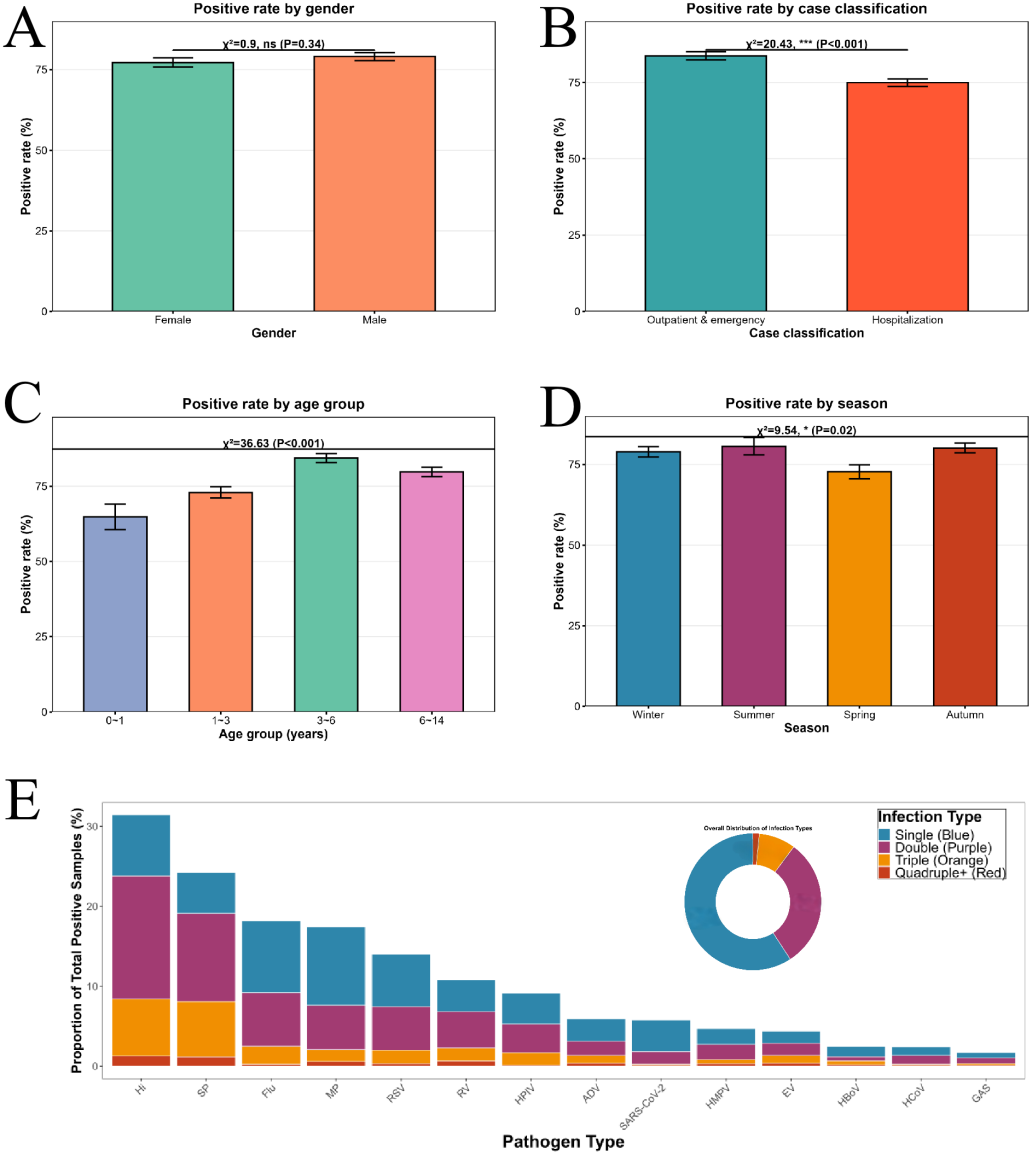
Pathogen detection rates and pathogen combinations by category. **(A)** Shows pathogen detection rates by gender and tests for differences; **(B)** shows pathogen detection rates by specimen source and tests for differences; **(C)** shows pathogen detection rates by age group and tests for differences; **(D)** shows pathogen detection rates by season and tests for differences; **(E)** shows a stacked bar chart of different pathogen infection combinations and a pie chart of overall infection rates.

This study included a total of 1,961 participants, with 1,534 (78.23%) testing positive for pathogens. Analysis by gender showed 1,089 males (detection rate 79.06%, 861/1,089) and 872 females (detection rate 77.18%, 673/872); the difference in detection rates between males and females was not statistically significant (χ² = 0.90, P > 0.05). There were statistically significant differences in detection rates across different age groups, seasons, and case classifications (χ² values of 38.22, 9.54, and 20.43, respectively, all P < 0.05): the detection rate was highest among children aged 3–6 years (84.30%) and during the summer (80.65%); the detection rate among outpatient and emergency department cases (83.69%) was higher than that among inpatients (74.90%). For detailed information, see **Figure 1**.

In this study, single infections and dual infections were the primary forms of respiratory pathogen infection, with single infections accounting for the highest proportion, representing 59.19% of positive samples. The tendency toward co-infection varies among different pathogens: SARS-CoV-2, MP, and HBoV were predominantly single infections (accounting for 68.54%, 56.18%, and 52.63%, respectively), with a relatively weak tendency toward co-infection; in contrast, SP and Hi had the lowest proportions of single infections (only 21.24% and 24.27%, respectively), with dual and triple infections accounting for over 70% of cases, making them the primary pathogens in mixed infections; EV had the highest proportion of quadruple or higher infections (8.96%) and was more likely to be involved in extreme multi-pathogen co-infections; HCoV, on the other hand, primarily presented as dual co-infections (45.95%). Overall, the incidence of extreme multi-pathogen co-infections (quadruple or higher) was low, with none exceeding 10%. See **Figure 1**.

### 3.2 Pathogen detection in different age groups and seasons

**Figure 2:**
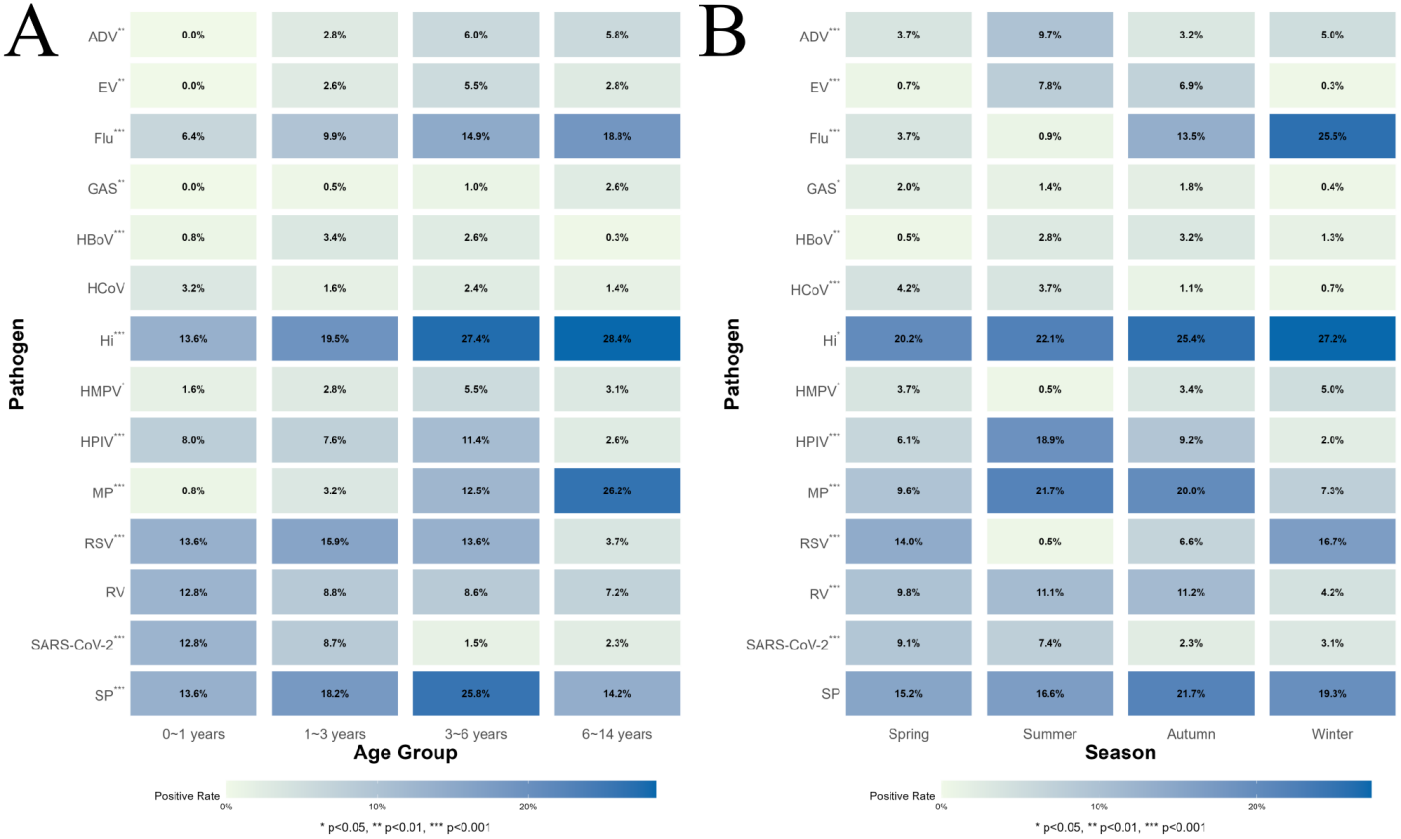
The detection status of different pathogens in different seasons and age groups. **(A)** Shows the detection rates of different pathogens across different age groups, as well as statistical analysis of variations in detection rates for the same pathogen across different age groups; **(B)** shows the detection rates of different pathogens across different seasons, as well as statistical analysis of variations in detection rates for the same pathogen across different seasons.

The prevalence of 14 pathogens in ARI cases varied across three age groups. Among these, there were no statistically significant differences in the prevalence of GAS, RV, and HCoV across the different age groups (P > 0.05), whereas statistically significant differences were observed in the prevalence of other pathogens across the different age groups (P < 0.05). See **Figure 1**. In the 0–1-year-old age group, the top three pathogens by positivity rate were: SP (13.6%), RSV (13.6%), and Hi (13.6%); in the 1–3-year-old age group, the top three pathogens were: Hi (19.5%), SP (18.2%), and RSV (15.9%); in the 3–6-year-old age group, the top three pathogens were: Hi (27.4%), SP (25.8%), and Flu (14.9%); in the 6–14-year-old age group, the top three pathogens were: Hi (28.4%), MP (26.2%), and Flu (18.8%). See **Figure 2**.

The prevalence of the 14 pathogens varied across different seasons. There was no statistically significant difference in SP prevalence across different age groups (P > 0.05), while there were statistically significant differences in the prevalence of other pathogens across different age groups (P < 0.05). The three pathogens with the highest prevalence in the fall were Hi (25.4%), SP (21.7%), and MP (20.0%); in spring, Hi (20.1%), SP (15.2%), and RSV (14.0%); in summer, Hi (22.1%), MP (21.7%), and HPIV (18.9%); and in winter, Hi (27.2%), Flu (25.5%), and SP (19.3%). See **Figure 2**.

### 3.3 Overview of daily case counts

**Figure 3:**
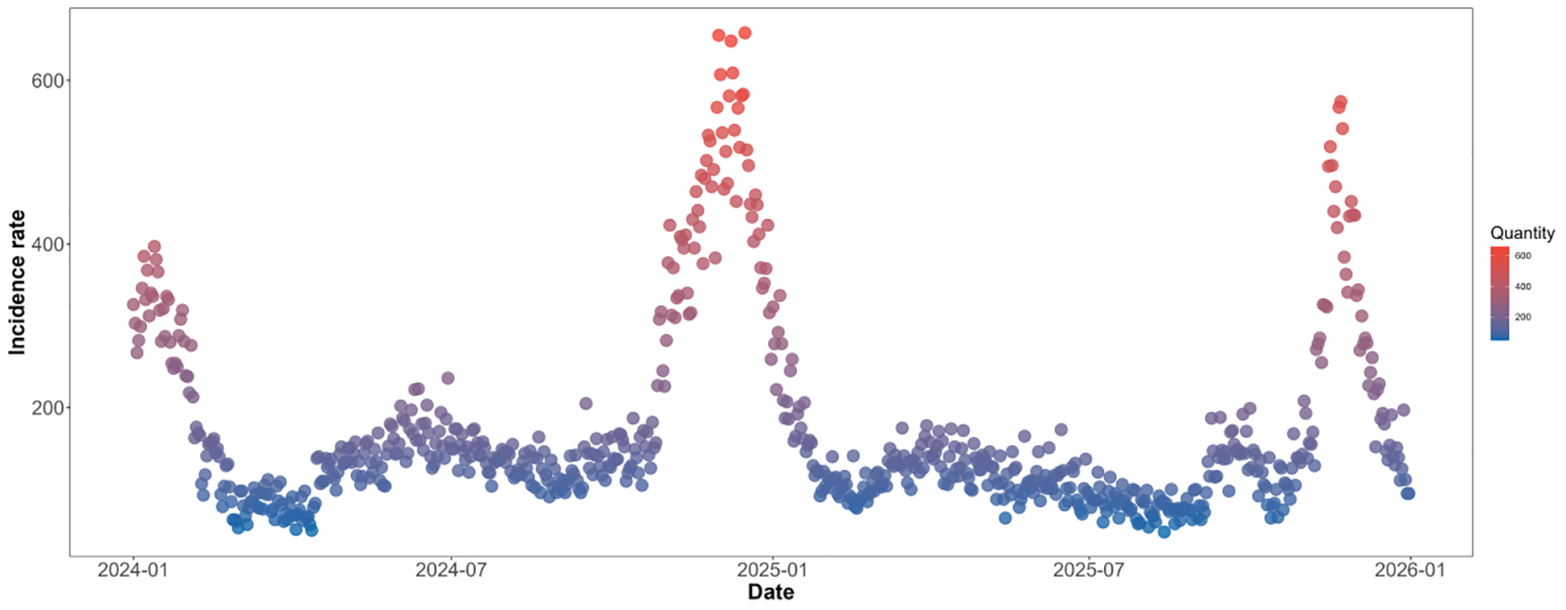
Daily incidence of acute respiratory infections (ARI) among children aged 0–14 in Shijiazhuang, 2024–2025.

In terms of temporal distribution, the daily number of cases exhibits distinct seasonal fluctuations and non-Poisson distribution characteristics: three peaks occurred—at the beginning of 2024, from late 2024 to early 2025, and from late 2025 to early 2026—with the peak from late 2024 to early 2025 being the highest, exceeding 700 cases per day; During the remaining periods, the number of cases was mostly concentrated in the 100–200 range, with relatively smooth fluctuations. However, during peak periods, the number of cases was significantly higher than the mean and exhibited high variability, further confirming the right-skewed distribution and non-Poisson characteristics of the data. This does not align with the assumptions of a Poisson distribution, which posits that “the mean and variance are approximately equal, and fluctuations are stable.” See **Figure 3**.

### 3.4 Variable filtering and description

**Figure 4:**
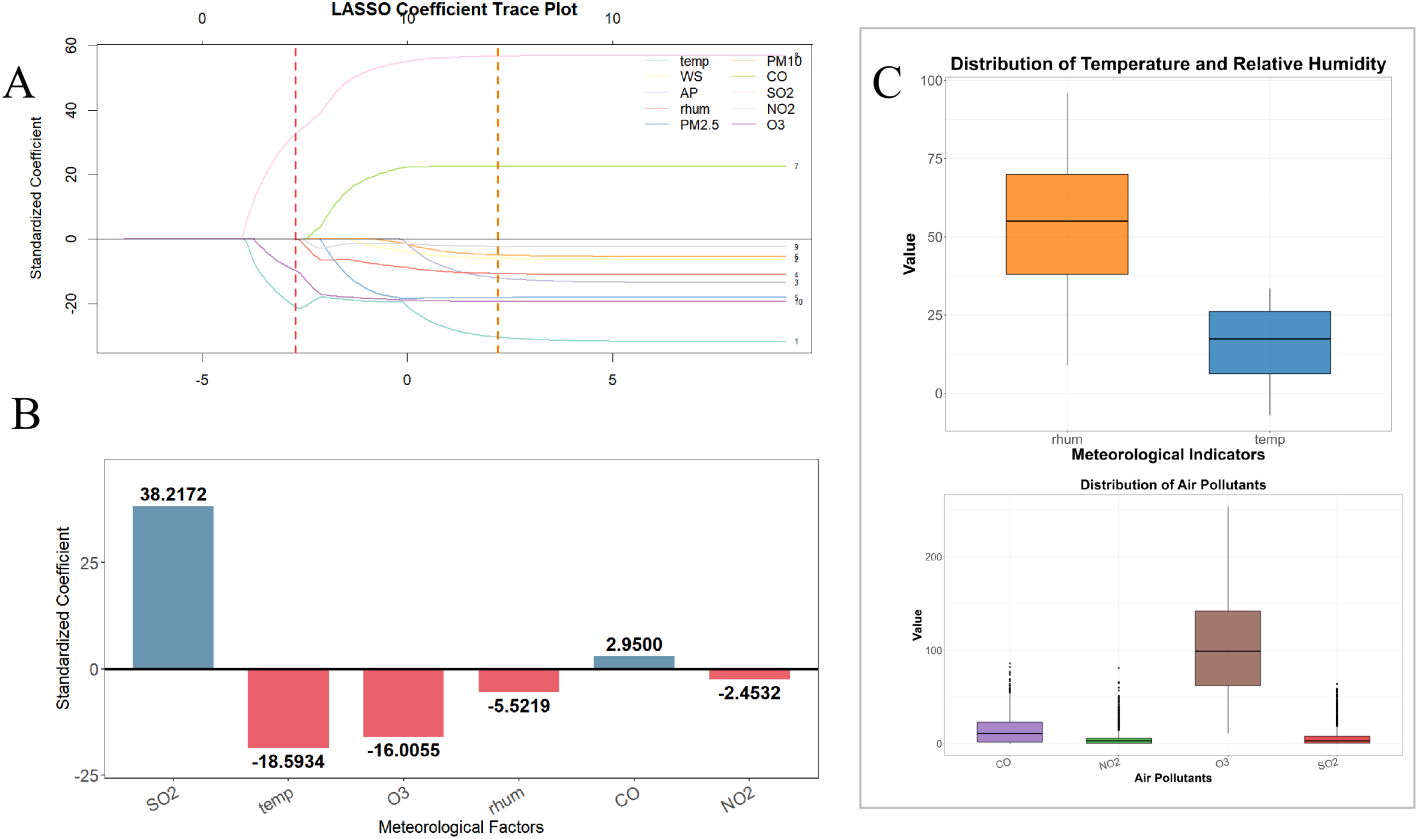
Screening and description of meteorological factors. **(A)** Shows the coefficient plot for LASSO regression; **(B)** shows a bar chart of the standardized coefficients for the core variables selected by LASSO regression; **(C)** shows a box plot describing the meteorological factors.

A LASSO regression model was used to screen variables related to meteorological and pollutant indicators. The optimal value of λ was determined by plotting the trajectory of the standardized coefficients as a function of the regularization parameter (log(λ)). Ultimately, six indicators were identified as significantly correlated with the outcome: SO_2_, temp, O_3_, rhum, NO_2_, and CO. The bar chart of standardized coefficients shows that SO_2_ (38.22) and CO (2.95) are positive influencing factors, with SO_2_ exhibiting the strongest positive effect; temperature (-18.59), O_3_ (-16.01), relative humidity (-5.52), and NO_2_ (-2.45) are negative influencing factors, with temperature having the most pronounced negative inhibitory effect. These results identify core environmental variables with stable predictive power for the outcome while controlling for multicollinearity. See **Figure 4**.

Normality tests revealed that all indicators had P < 0.001, indicating a non-normal distribution; therefore, median (interquartile range) was used for statistical description. Air temperature was 17.40 (6.25, 26.15) °C; relative humidity was 55.00 (38.00, 70.00)%. Regarding pollutant indicators: CO was 11.00 (1.80, 23.00) mg/m³, SO_2_ was 3.00 (0.80, 8.00) μg/m³, NO_2_ was 3.00 (0.60, 6.00) μg/m³, and O_3_ was 99.00 (62.50, 142.00) μg/m³. See **Figure 4**.

### 3.5 The relationship between meteorology and ARI

**Figure 5:**
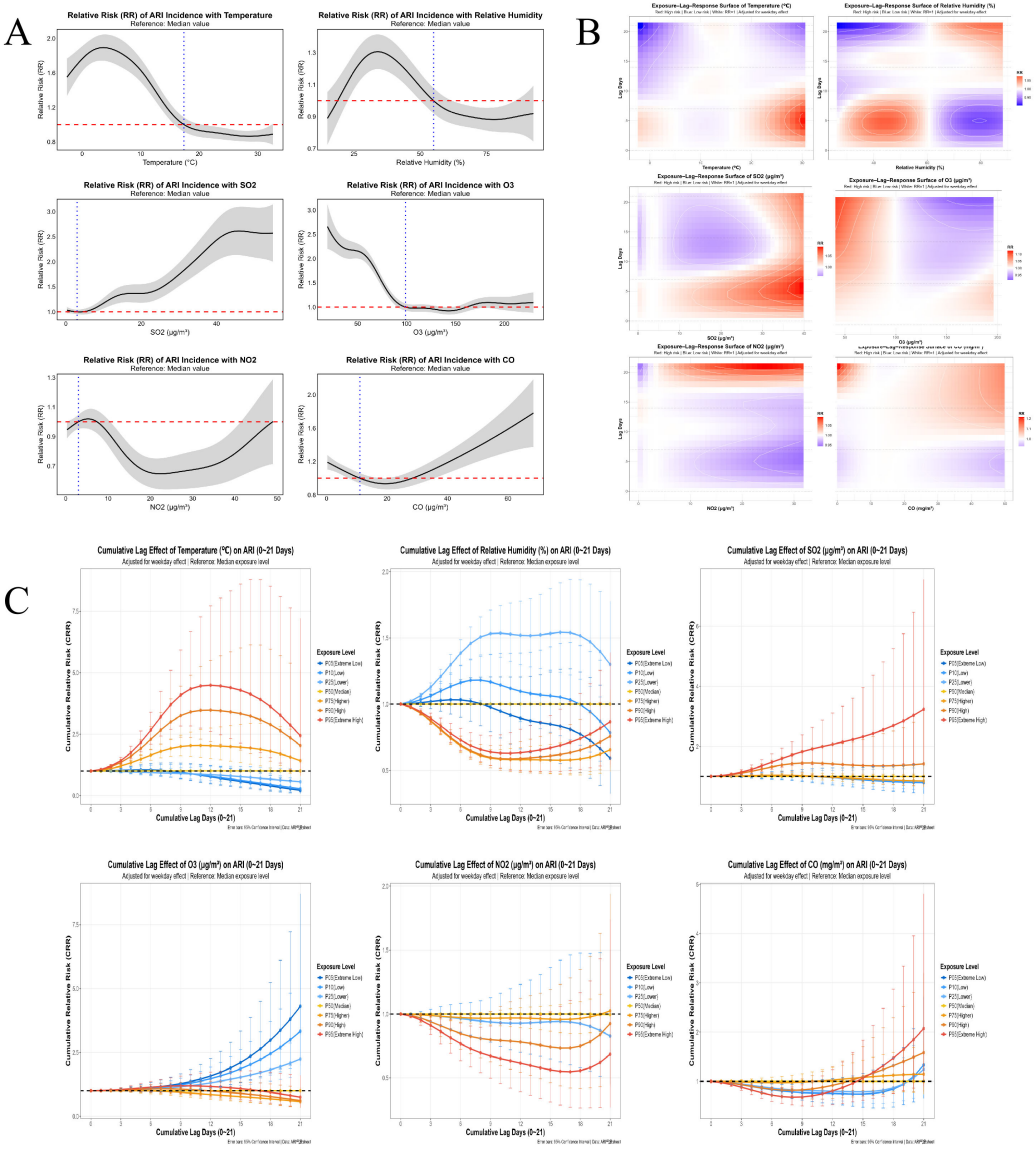
The relationship between various meteorological factors and ARI. **(A)** Shows the relative risk changes of various meteorological factors and ARI based on GAM; **(B)** shows the 3D heatmap of the exposure-lag-effect relationship between various meteorological factors and ARI based on DLNM; **(C)** shows the quantile plot of the single-day lag effects of various meteorological factors on ARI based on DLNM.

All factors showed a nonlinear association with the incidence of ARI. The maximum relative risk (Max RR) for each factor and the corresponding exposure levels are as follows: the risk of ARI was highest at an air temperature of 3.63 °C, with an RR of 1.89 (95% CI: 1.74–2.05); The risk of ARI incidence was highest at a relative humidity of 34%, with an RR of 1.31 (95% CI: 1.20–1.41); The risk of ARI incidence was highest at an SO_2_ concentration of 45.90 μg/m³, with an RR of 2.61 (95% CI: 2.13–3.10); The risk of ARI incidence was highest at an NO_2_ concentration of 5.28 μg/m³, with an RR of 1.02 (95% CI: 0.95–1.09); The risk of ARI was highest at a CO concentration of 68.4 μg/m³, with an RR of 1.78 (95% CI: 1.38–2.19); the risk was highest at an O_3_ concentration of 19.20 μg/m³, with an RR of 2.66 (95% CI: 2.20–3.12). Overall, O_3_ and SO_2_ had the strongest pathogenic effects on ARI incidence. For further details, see SO_2_ exposure had a significant concentration-dependent, lagged, and cumulative effect on the incidence of acute respiratory infections (ARI): the overall effect of low-concentration P10 (0.4 μg/m³) was weak, with only a slight increase observed after a 7-day lag (RR = 1.0287); at medium-to-high concentrations, the risk gradually increased with rising SO_2_ levels; at the high-concentration P90 (29.9 μg/m³), the cumulative effect peaked after a 10-day lag (RR = 1.4477), with a significant increase in the risk of incidence; the 3D risk surface indicates that the highest short-term risk occurred at 39.4 μg/m³ with a 6-day lag (RR = 1.0913); while the overall cumulative lag results further indicate that high SO_2_ concentrations of 39.4 μg/m³ result in the highest risk of incidence (RR = 3.2375). Overall, the pattern is such that higher concentrations, longer lag times, and stronger cumulative effects lead to a more pronounced increase in ARI incidence. See **Figure 5**.

Temperature exerts a nonlinear effect on the incidence of acute respiratory infections (ARI), with a significant increase at high temperatures and a negligible effect at low temperatures: the highest risk on the 3D effect surface was observed at 30.6 °C with a 5-day lag (RR = 1.2288), while the highest overall cumulative risk was observed at 31.1 °C (RR = 2.4405); The P10/P25/P50 low-to-moderate temperature groups showed minimal impact, while the P75/P90 high-temperature groups (26.1 °C and 29.6 °C) exhibited a substantial increase in risk, with the P90 cumulative effect peaking at a 12-day lag (RR = 3.4777). Overall, the pattern indicates that as temperature increases, the lag lengthens, the cumulative effect strengthens, and the rise in incidence becomes more pronounced. See **Figure 5**.

Relative humidity exerts a nonlinear influence on the incidence of ARI, with increased risk at low to moderate humidity levels and no significant risk at high humidity levels. The 3D effect surface indicates the highest risk at 39.0% relative humidity with a 5-day lag (RR = 1.0741), and the overall cumulative effect reaches a peak risk of 42.5% (RR = 1.3767); The risks at the low-to-moderate humidity quantiles P10 (27.0%) and P25 (38.0%) increased significantly, with the P25 quantile reaching a peak at a cumulative lag of 16 days (RR = 1.5424) and a single-day lag of 5 days (RR = 1.0737), while the risks at the high-humidity quantiles P50 (55.0%), P75 (70.0%), P90 (80.0%) were all close to baseline levels (RR ≈ 1.0). Overall, the risk of ARI incidence was highest in the 27%–42.5% low-to-moderate humidity range, and the risk further increased with extended lag times. See **Figure 5**.

O_3_ exhibits a unique influence on ARI incidence, characterized by high risk at low concentrations and weaker risk at high concentrations. The 3D risk surface indicates that the highest risk occurs at 33.5 μg/m³ with a 21-day lag, yielding an RR of 1.1317; The overall cumulative effect further indicates that 33.5 μg/m³ is the globally highest-risk concentration, with an RR as high as 4.3051; the P90 high concentration (177.0 μg/m³) only shows a slight increase in risk with a lag of 4–7 days, with the highest RR for single-day and cumulative lags being 1.0153 and 1.0772, respectively; while the P75 concentration (142.0 μg/m³) showed an even weaker risk. Overall, the results indicate that long-term cumulative exposure to low-concentration ozone poses a significant risk, whereas high-concentration ozone has a limited impact on ARI incidence. See **Figure 5**.

The impact of NO_2_ on the incidence of ARI is characterized by a prominent long-term delayed risk at high concentrations and a moderate increase in risk. The core patterns are as follows: the 3D effect surface shows that the highest risk occurs at 25.4 μg/m³, with a 21-day lag (RR = 1.1109). The P90 high concentration (18.9 μg/m³) also peaks in risk after a 21-day lag (RR = 1.1024), consistent with the pattern of long-term cumulative exposure risk; In the overall cumulative effect, 6.8 μg/m³ represents the highest global risk concentration (RR = 1.0261), while the P75 (5.8 μg/m³) cumulative risk with a 21-day lag has an RR of 1.0253, indicating a similar risk level; Overall, the risk impact of NO_2_ on ARI is primarily characterized by high concentrations (18.9–25.4 μg/m³) with a long-term lag (21 days). See **Figure 5**.

The impact of CO on the incidence of ARI exhibits a dual characteristic: the highest overall risk at high concentrations and a significant long-term lag risk at low concentrations. The core patterns are as follows: The overall cumulative effect shows that 49.40 mg/m³ is the concentration with the highest overall risk, with an RR of 2.0726, representing a doubling of risk compared to the baseline level; The risk associated with high-concentration quantiles significantly accumulates as the lag time increases; for the P90 (38.90 mg/m³), the RR reaches 1.5840 after a cumulative lag of 21 days, and 1.0648 after a single-day lag of 17 days; for the P75 (22.90 mg/m³), the RR is 1.1458 after a cumulative lag of 21 days; At the same time, the 3D risk surface revealed that low concentrations (0.40 mg/m³) also posed a significant risk after a 21-day lag (RR = 1.2173). Overall, the harm of CO to ARI is primarily characterized by long-term cumulative exposure (21 days) at high concentrations (38.90–49.40 mg/m³), and long-term exposure to low concentrations also affects health. See **Figure 5**.

## 4 Discussion

This study analyzed the prevalence of 14 respiratory pathogens in Shijiazhuang in 2024. The overall detection rate was 78.23%, which was higher than the previously reported detection rate (46.43%) among hospitalized patients with acute respiratory infections (ARTIs) in Shijiazhuang (Zheng et al., 2024), as well as higher than the rates in Guangdong Province (36.4%) (Li et al., 2020), Gansu Province (65.6%) (Shi and Wei, 2022), and the overall detection rate of multiple respiratory pathogens in nine Chinese provinces, including Anhui (40.59%) (Cui et al., 2022). It is also higher than rates in Italy (50.44%) (De Conto et al., 2019) and the United States (51.9%–67.8%) (Timbrook et al., 2024). Differences in detection rates across regions may be related to factors such as the types of pathogens tested, case composition, geography, and the year of testing (Li et al., 2021). The results of this study show that the detection rates of AP, Hi, and Flu were relatively high, differing from reports from Guangdong (Li et al., 2020) and Henan (Wan et al., 2024), but similar to the findings of another study in Henan (Seng et al., 2025). This is related to variations in the pathogens circulating in different years and regions, as well as the scope or methodology of the selected studies. The number of ARI cases in Shijiazhuang exhibits a bimodal pattern, with a major peak from December to January of the following year and a minor peak in May–June. The former is attributed to winter’s low temperatures prolonging the survival of pathogens such as Flu and RSV, compounded by poor ventilation in crowded indoor settings and increased population mobility before the Spring Festival, which heighten transmission risks. The latter is closely related to the resurgence of viral activity—such as RV and ADV—as temperatures rise in late spring, as well as increased transmission through contact in group settings following children’s return to school (Zheng et al., 2024). See **Table 1**.

**Table 1:** Detection results for the sample population.

Category	Positive cases	Negative numbers	Quantity	Positive rate(%)	*P*
Sex					0.34
Male	861	228	1089	79.06	
Fremale	673	199	872	77.18	
Age Group(year)					<0.01
0~1	81	44	125	64.80	
1~3	412	153	565	72.92	
3~6	520	97	617	84.28	
6~14	521	133	654	79.66	
Season					0.02
Spring	296	111	407	72.73	
Winter	539	144	683	78.92	
Autumn	524	130	654	80.12	
Summer	175	42	217	80.65	
Source of Specimens					<0.01
Outpatient	913	306	1219	74.90	
Inpatient	621	121	742	83.69	

The rate of single infections (46.30%) was higher than that of mixed infections (23.97%), and the difference between the proportions of single infections (59.19%) and mixed infections (40.81%) among positive samples was statistically significant (χ² = 51.84, P < 0.05). which is consistent with the common pattern of respiratory infections: following a primary infection with a single pathogen, the immune system prioritizes a targeted response to suppress invasion by other pathogens, whereas mixed infections require multiple pathogens to breach the immune defenses, making them relatively less likely to occur (Cheemarla et al., 2021). In terms of the ranking of detected pathogens, Flu, Hi, and SP ranked in the top three, which is similar to the results of studies conducted in many regions within China (Wan et al., 2024; Yuan et al., 2024), among which MP, due to its lack of a cell wall, ease of colonization in the respiratory tract, and resistance to β-lactam antibiotics, is highly transmissible among children and adolescents (Oishi et al., 2025). Hi and SP, as normal respiratory colonizers, can easily become pathogenic when host immunity is compromised (e.g., damage to the mucosal barrier following viral infection); children, in particular, with their immature immune systems, constitute a high-risk population for these two types of pathogens (Nelson et al., 2016). In mixed infection patterns, 10 pathogens including Hi and SP all exhibited a higher prevalence of multiple infections compared to single infections. Dual infections were predominantly MP+Hi combinations, while triple infections were most commonly MP+SP+Hi combinations. This phenomenon is closely related to synergistic interactions among pathogens: MP infection first disrupts the respiratory epithelial cell barrier, creating conditions for the adhesion and colonization of Hi and SP, while viral infections such as RV and ADV further suppress immune function, exacerbating the risk of secondary pathogen infections, thereby forming dominant “virus-bacteria” or “bacteria-bacteria” synergistic infection combinations. From a clinical perspective, the findings of this study provide important evidence for the precise diagnosis and treatment of respiratory infections—the high detection rate suggests that clinicians should prioritize pathogen testing to determine the cause, while the predominant combinations of mixed infections require treatment regimens that address multiple pathogens (e.g., MP+Hi infections require the combined use of macrolides and antibiotics sensitive to Hi) (Nelson et al., 2016; Oishi et al., 2025).

Differences in the age and seasonal distribution of ARI pathogens are primarily determined by the development of population immunity, pathogen characteristics, and environmental conditions. In terms of age, MP, Hi, and influenza are most prevalent among 7- to 14-year-olds. Since MP lacks a cell wall, it spreads easily via droplets in schools, and adolescents lack stable immunity against it; Hi causes disease through cross-infection in crowded settings (Kappler et al., 2024); SP and RV are most prevalent among children aged 3–6 years, as their immune systems and mucosal barriers are not yet fully developed, and frequent contact in daycare settings allows opportunistic pathogens like SP to breach these defenses; RSV and SARS-CoV-2 are most prevalent among infants and toddlers aged 0–3 years, as their immune systems and cilia function are weak, RSV exhibits strong tropism for their respiratory epithelial cells, and SARS-CoV-2 takes advantage of the lack of innate immune memory in this age group (Cheemarla et al., 2021). Only GAS and HBoV show no age-related differences; GAS spreads widely via skin and mucous membranes, while HBoV has a long latent infection period, resulting in similar exposure risks across all age groups (Nelson et al., 2016). Seasonally, pathogens exhibit distinct patterns: MP and Hi peak in the fall, as dropping temperatures weaken mucosal resistance and dry conditions facilitate Mycoplasma transmission; SP, Flu, and Hi peak in the winter, as low temperatures prolong pathogen survival, and poor ventilation in crowded indoor settings exacerbates transmission; MP, HPIV, and EV peak in the summer, as high temperatures and humidity support EV survival, and increased water exposure among children leads to contact transmission; SARS-CoV-2 and HCoV are most prevalent in spring, as rising temperatures increase population mobility and restore pathogen activity. Overall, the prevalence of respiratory pathogens results from the interaction between host, pathogen, and environment. Differences in age-related immunity determine susceptibility stratification, while seasonal and environmental changes regulate the efficiency of pathogen transmission, providing a basis for targeted prevention and control. See **Table 2**.

**Table 2:** Detection rates of pathogens across different age groups and tests for differences.

Category	0~1 years(n=125)		1~3 years(n=565)		3~6 years(n=617)		6~14 years(n=654)	
	Positive cases	Positive rate(%)	Positive cases	Positive rate(%)	Positive cases	Positive rate(%)	Positive cases	Positive rate(%)
SARS-CoV-2*	16	12.8	49	8.67	9	1.46	15	2.29
Flu*	8	6.4	56	9.91	92	14.91	123	18.81
RSV*	17	13.6	90	15.93	84	13.61	24	3.67
ADV*	0	0	16	2.83	37	6	38	5.81
HMPV*	2	1.6	16	2.83	34	5.51	20	3.06
HPIV*	10	8	43	7.61	70	11.35	17	2.6
HCoV	4	3.2	9	1.59	15	2.43	9	1.38
HBoV*	1	0.8	19	3.36	16	2.59	2	0.31
RV	16	12.8	50	8.85	53	8.59	47	7.19
EV*	0	0	15	2.65	34	5.51	18	2.75
MP*	1	0.8	18	3.19	77	12.48	171	26.15
GAS*	0	0	3	0.53	6	0.97	17	2.6
SP*	17	13.6	103	18.23	159	25.77	93	14.22
Hi*	17	13.6	110	19.47	169	27.39	186	28.44

*indicates a significant difference (*P* < 0.05) in the same pathogen across different age groups.

Weather conditions and air pollutants influence the risk of acute respiratory infections (ARIs) by affecting respiratory tract defenses and regulating the survival and transmission of pathogens. Among meteorological factors, low temperatures weaken ciliary movement in the mucous membranes and prolong the survival of pathogens such as influenza, while poor ventilation in crowded indoor settings exacerbates transmission (Kilgour et al., 2004); low humidity causes dehydration and damage to the mucous membranes, and dry air facilitates the suspension and spread of droplet nuclei (Edwards et al., 2025; Huang et al., 2025). Among pollutants, high SO_2_ reacts with mucosal moisture to form acidic substances, damaging the mucosa and inhibiting macrophage phagocytosis (Sun et al., 2025); low O_3_ concentrations penetrate deep into the alveoli, inducing oxidative stress and inflammation (Ghosh, 2024); high NO_2_ causes mucosal congestion and edema, reducing defenses and impairing immune cell function (von Nieding and Wagner, 1979). See **Table 3**.

**Table 3:** Detection rates of various pathogens across different seasons and tests for differences.

Category	Autumn(n=654)		Spring(n=407)		Summer(n=217)		Winter(n=683)	
	Positive cases	Positive rate(%)	Positive cases	Positive rate(%)	Positive cases	Positive rate(%)	Positive cases	Positive rate(%)
SARS-CoV-2*	15	2.29	37	9.09	16	7.37	21	3.07
Flu*	88	13.46	15	3.69	2	0.92	174	25.48
RSV*	43	6.57	57	14	1	0.46	114	16.69
ADV*	21	3.21	15	3.69	21	9.68	34	4.98
HMPV*	22	3.36	15	3.69	1	0.46	34	4.98
HPIV*	60	9.17	25	6.14	41	18.89	14	2.05
HCoV*	7	1.07	17	4.18	8	3.69	5	0.73
HBoV*	21	3.21	2	0.49	6	2.76	9	1.32
RV*	73	11.16	40	9.83	24	11.06	29	4.25
EV*	45	6.88	3	0.74	17	7.83	2	0.29
MP*	131	20.03	39	9.58	47	21.66	50	7.32
GAS*	12	1.83	8	1.97	3	1.38	3	0.44
SP	142	21.71	62	15.23	36	16.59	132	19.33
Hi*	166	25.38	82	20.15	48	22.12	186	27.23

*indicates a significant difference (*P* < 0.05) in the same pathogen across different age groups.

This study employed GAM and DLNM to investigate the impact of environmental factors on ARI incidence. The two models yielded differing conclusions regarding the effect of temperature: GAM suggested that rising temperatures reduce the risk of ARI, whereas DLNM, after incorporating lag and cumulative effects, clearly indicated that high temperatures increase the risk of ARI, with the risk intensifying as temperatures rise and lag times lengthen. This discrepancy does not stem from a structural error in the models but rather reflects a distinctive characteristic of the study region. The root cause lies in the differing focuses and perspectives of the two models: GAM emphasizes the immediate effects of temperature on ARI incidence, whereas DLNM places greater emphasis on the cumulative effects of temperature. This suggests that when investigating the association between meteorological factors and disease, it is necessary to simultaneously consider the influences of lag effects, cumulative effects, and immediate effects.

All environmental factors exhibit a nonlinear association with ARI incidence, and their pathogenic effects show significant heterogeneity. O_3_ and SO_2_ have the strongest pathogenic effects and are key risk factors; NO_2_ has a weak pathogenic effect and limited independent impact; temperature, relative humidity, and CO all have high-risk exposure windows, providing support for the delineation of risk thresholds. The impact of SO_2_ on ARI is concentration-dependent, exhibits lag effects, and is cumulative; medium-to-high concentrations and long-term cumulative exposure significantly amplify the risk of incidence (Sun et al., 2025). High temperatures are a major meteorological trigger for ARI, while low and moderate temperatures have minimal effects. Under high temperatures, the risk of ARI increases as the lag period lengthens and cumulative effects intensify, highlighting the importance of prevention and control during summer heatwaves. The impact of relative humidity on ARI is characterized by increased risk at low to moderate humidity levels and no significant effect at high humidity levels; low to moderate humidity constitutes a high-risk window, and its long-term lag effect can serve as a reference for intervention strategies. O_3_ exerts a unique influence on ARI; long-term cumulative exposure to low concentrations has a significant pathogenic effect, while exposure to high concentrations has limited impact, making it the environmental factor with the most pronounced pathogenic effect (Ghosh, 2024). The pathogenic effect of NO_2_ on ARI is mild; the core issue is that high concentrations and long-lag exposure pose a prominent risk, but its independent contribution is limited, and controlling its concentration alone yields poor prevention and control results (von Nieding and Wagner, 1979). CO poses a dual threat to ARI; long-term exposure to both low and high concentrations increases the risk of disease (Sun et al., 2025), and both must be included in air quality management. In summary, ARI incidence is jointly influenced by meteorological factors and atmospheric pollutants, with each factor characterized by non-linearity, lag, and cumulative effects; O_3_ and SO_2_ are key causative factors. DLNM is better able to comprehensively reveal the health effects of environmental exposure, providing a scientific basis for the precise prevention and control of ARI.

This study has certain limitations; it did not clarify the long-term trends in pathogen prevalence or the long-term dynamic effects of meteorological factors, nor did it thoroughly investigate the underlying mechanisms of mixed infections. Future research could involve long-term follow-up monitoring to clarify the causal relationships among various factors, and combine *in vitro* experiments with clinical follow-up data to explore the mechanisms of mixed infections, thereby providing support for clinical diagnosis and treatment.

## 5 Conclusions

The overall detection rate of respiratory pathogens in Shijiazhuang is high, with MP, Hi, and SP being the predominant pathogens. “MP + bacteria” is the primary type of mixed infection, and regional variations in detection rates are influenced by multiple factors, including testing conditions. ARI incidence exhibits clear age stratification and seasonal patterns; therefore, differentiated prevention and control measures should be developed for different age groups and seasons, with particular attention to the winter peak. Low temperatures or prolonged periods of high temperatures, low humidity, and high SO_2_ concentrations are key environmental risk factors, exhibiting a dual “immediate + cumulative” effect. By clarifying the pathogen spectrum, distribution patterns, and environmental risks, this study provides data support for the precise diagnosis and treatment of ARI, as well as for population- and season-specific prevention and control measures and environmental risk warnings, offering valuable references for the management of respiratory diseases in the local area.

## Data Availability

The relevant data cannot be provided directly. If needed, please contact the Hebei Provincial Center for Disease Control and Prevention, China.
